# Combined Effects of Carrageenan and Konjac Gums on the Physicochemical Properties of a Plant-Based Smoked Salmon Analog

**DOI:** 10.3390/foods14213793

**Published:** 2025-11-05

**Authors:** Silvia Mata, Juan I. Maté, Ignacio Angós

**Affiliations:** Department of Agronomy, Biotechnology and Food, Institute for Sustainability & Food Chain Innovation—ISFOOD, Universidad Pública de Navarra (UPNA), Campus de Arrosadia s/n, 31006 Pamplona, Navarra, Spain; mata.161023@e.unavarra.es (S.M.); juan.mate@unavarra.es (J.I.M.)

**Keywords:** plant-based seafood, smoked salmon analog, hydrocolloids, carrageenans, konjac gum, textural properties, rheological analysis

## Abstract

This study aimed to develop and characterize plant-based smoked salmon analogs by evaluating the effects of three hydrocolloids (kappa carrageenan, iota carrageenan, and konjac gum) on their physicochemical, textural, and functional properties. Sixteen formulations were prepared by varying the proportions of these gelling agents while maintaining a constant base formulation. Water-binding capacity (WBC), pH, hardness, springiness, viscoelastic moduli (elastic and viscous), sliceability, and layer definition were analyzed. Kappa carrageenan significantly increased firmness and improved slicing properties. Iota carrageenan enhanced springiness and structural definition. Although konjac gum improved moisture retention, high concentrations of it reduced structural integrity. Multivariate analyses, including principal component analysis (PCA) and hierarchical clustering (HCA), revealed an inverse relationship between mechanical properties (hardness and moduli) and WBC, while springiness formed an independent variable cluster. The first two components accounted for 72.52% of total variance; indicated by the PCA. Among the sixteen formulations, three stood out: T01 exhibited the highest firmness and sliceability; T02 presented excellent elasticity and moisture retention; and T16 achieved a well-balanced profile, closely resembling commercial smoked salmon in both texture and appearance. These findings demonstrate that tailored hydrocolloid combinations can effectively mimic the texture and appearance of smoked salmon, providing a novel formulation strategy for next-generation plant-based seafood alternatives.

## 1. Introduction

In recent years, consumers have reduced seafood consumption due to growing environmental, ethical, and health concerns. Overfishing, biodiversity loss, and ecosystem degradation, together with the environmental impact of aquaculture and animal welfare issues, have intensified the search for sustainable alternatives [1].

Contamination of fish with microplastics and heavy metals remains a concern. Microplastics may cause oxidative stress, tissue damage, and immune disruption in both fish and humans, although their full impact is still under investigation [2,3]. Heavy metals, such as mercury, accumulate in aquatic ecosystems and concentrate in predatory species such as tuna, posing neurotoxic and reproductive risks, especially for vulnerable populations [4].

For the reasons above-mentioned, the search for sustainable food alternatives capable of meeting the global demand for fish products without threatening marine biodiversity has become increasingly important. In this context, plant-based fish analogs have emerged as a promising strategy to reduce pressure on the oceans while satisfying the needs of consumers wishing to decrease their intake of animal-derived foods. As a result, plant-based meat and fish analogs are gradually evolving from niche products into mainstream consumer choices [5]. The commercial success of such products not only depends on their environmental impact and ethical value, but also on consumer perception and acceptance. This has encouraged the development of research focused on assessing sensory attributes and evaluating the market viability of these products [6].

Despite the growing demand, plant-based alternatives to fish and seafood still represent a very small proportion of the overall market. Nevertheless, investments and sales in this sector have increased rapidly in recent years, reflecting a wider effort to address this emerging consumer demand [1]. One of the main challenges facing plant-based fish substitutes is replicating the characteristic texture of fish, which is defined by a specific balance of elasticity and disintegration during chewing. However, many companies concentrate their efforts on reproducing the appearance and flavor of seafood, while often overlooking structural and textural fidelity.

A promising strategy to overcome this challenge involves the use of plant proteins in combination with polysaccharides, which function as hydrocolloids capable of forming gel-like networks that mimic the consistency and mouthfeel of conventional fish flesh [7]. Hydrocolloids are long-chain polymers that are partially or fully soluble in water and have the capacity to form gels by thickening and stabilizing emulsions. In the food industry, they are widely employed due to their rheological and texturizing properties, contributing to improved sensory characteristics in a wide variety of products [8]. In addition to their technological roles, some hydrocolloids can promote satiety by delaying gastric emptying and positively influence the gut microbiota by supporting the growth of beneficial bacteria and the production of short-chain fatty acids [9,10].

Hydrocolloids are generally classified by their origin into the following categories: natural (e.g., extracted from algae or plants), modified or semi-synthetic, and synthetic [8]. Despite requiring higher concentrations for similar effects, the interest in natural ingredients is increasing, compared to the more expensive synthetic hydrocolloids; even though the synthetics are more efficient [8,10].

In food applications, hydrocolloids are used as thickeners, gelling agents, stabilizers and encapsulating agents. They also function as fat replacers in low-calorie products. Accordingly, they produce the desired viscosity and texture without negatively affecting sensory perception. Their uses also extend to other sectors such as cosmetics and pharmaceuticals, where they are employed to create protective films and coatings. For their use in food applications, hydrocolloids must comply with safety regulations and labelling standards and must be recognized as GRAS (Generally Recognized as Safe) substances [10].

Konjac glucomannan (KGM) is a water-soluble hydrocolloid extracted from the tubers of Amorphophallus konjac. It is valued for its health benefits including the prevention of diabetes, obesity and hyperglycemia [11]. Its structure includes D-glucose and D-mannose monomers with acetyl groups that, upon deacetylation under alkaline conditions, allow for the formation of irreversible elastic gels through hydrogen bonding [7]. KGM is GRAS-certified and widely used in low-fat food products such as mayonnaise, yoghurt and cheese to enhance texture and stability [12]. It is also used in sausage and restructured seafood formulations where it improves water retention, gel strength and cohesion; thus, enhancing sensory performance [7,13].

Carrageenans are natural polysaccharide hydrocolloids derived from red algae (Rhodophyceae), mainly composed of D-galactose and 3,6-anhydro-D-galactose units with sulfate groups as primary substituents. These compounds are widely used in the food industry for their gelling, thickening, stabilizing and water-retaining properties, as well as for their ability to interact with proteins [14].

There are three main types of carrageenans (kappa (κ), iota (ι), and lambda (λ)) with distinct structural and functional properties. Traditionally, they have been used in meat, dairy and bakery products. However, recent innovations have extended their use to edible coatings, plant-based analogs and 3D food printing applications [15,16,17].

Kappa carrageenan forms firm, brittle gels that contribute to a robust product structure; while iota carrageenan forms softer, elastic gels that retain moisture and confer a juicier mouthfeel [14,17]. Both types exhibit reversible helix-coil transitions; their combination can yield textures similar to those found in real smoked salmon [14,18].

Beyond hydrocolloids, achieving the desired sensory profile of a salmon analog requires the use of natural colorants (e.g., paprika, radish, carrot extracts or iron oxide) and flavorings to reproduce the characteristic orange tone and smoky taste of traditional smoked salmon. Maltodextrin is useful to stabilize emulsions with fat and thus improves the texture and mouthfeel of the final product [19]. The combination of these elements contributes to the authenticity of plant-based fish analogs in both appearance and flavor.

To address the gap in structural replication, this study introduces a novel formulation strategy that systematically explores the combined use of three hydrocolloids, kappa carrageenan, iota carrageenan, and konjac gum, at a fixed total concentration, aiming to replicate the texture and functionality of smoked salmon rather than focusing solely on visual or flavor aspects. This approach contributes to a better understanding of hydrocolloid interactions in plant-based seafood analogs and offers valuable insights for future innovation in the category.

In this study, a strategic combination of hydrocolloids with gelling, emulsifying and thickening capabilities (kappa carrageenan, iota carrageenan and konjac gum), which can replicate the structure and appearance of smoked salmon, is hypothesized to produce a fibrous, juicy and sensorially acceptable product. Kappa carrageenan is expected to contribute firmness, iota carrageenan to improve juiciness and moisture retention, while konjac is expected to enhance elasticity and cohesion; therefore, resulting in an optimized texture. The right balance of these components is anticipated to result in a formulation with superior sensory acceptance; consequently, outperforming single-ingredient or unbalanced mixtures [7,18,20].

Therefore, the objective of this research was to develop a vegan salmon analog that faithfully reproduces the texture, appearance and flavor of smoked animal salmon. This objective was achieved by optimizing the proportions of the three main hydrocolloids and assessing the resulting formulations in terms of their physicochemical, textural and rheological properties.

## 2. Materials and Methods

### 2.1. Materials

For the development of the 16 salmon analog formulations, pea protein with 82% protein content, supplied by Energy Feelings (Tarragona, Spain) was used. The kappa and iota carrageenan hydrocolloids were provided by Töufood (Barcelona, Spain), while konjac gum was supplied by Industrias Roko (Llanera, Spain). In addition, flavorings, colorants and maltodextrin were obtained from various national suppliers. The liquid phase of the formulations consisted of potable water at room temperature and refined sunflower oil, both purchased locally. Slices of smoked salmon from Norway, obtained from a local retailer, were used as the control sample. This control was tested using the same preparation and analytical protocols as the plant-based formulations, enabling consistent comparison across all parameters.

Note: Due to confidentiality agreements within the framework of this applied research project, detailed specifications of certain materials (e.g., molecular weight, purity, or product codes) cannot be disclosed. However, all ingredients used were food-grade, commercially available and selected based on established functionality.

### 2.2. Sample Preparation

**Figure 1:**
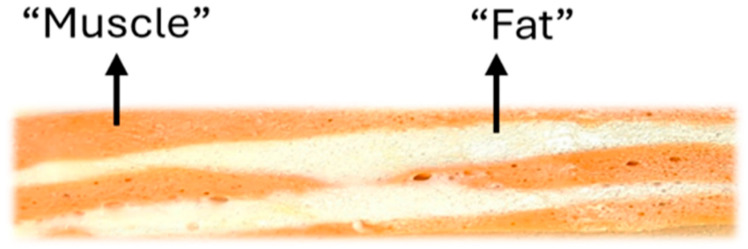
Structural layering of the plant-based salmon analog. Orange areas represent “lean” muscle-like layers, while white areas correspond to “fat-like” regions.

Sixteen vegan salmon analog formulations were developed: they consisted of two visually and functionally distinct parts: a “lean” phase (orange) and a fatty phase (white) (Figure 1).

The formulations varied only in the composition of the lean phase, as the fatty phase remained constant in both quantity and formulation. The lean formulations differed in the proportions of the three main gelling agents (kappa carrageenan, iota carrageenan and konjac gum) while maintaining a total hydrocolloid content of 3% (dry basis). The specific proportions used in each formulation are detailed in Table 1.

This experimental design was intended to evaluate the contribution of each hydrocolloid, alone or in combination, to the final texture and structure of the product. By keeping the total hydrocolloid content constant and systematically varying their individual proportions, it was possible to assess both the individual functionality and potential synergistic interactions between them under controlled conditions.

The remaining ingredients, including pea protein, maltodextrin, flavoring and colorants, were kept constant across all formulations. Water and oil were also standardized as liquid components. For both the lean and fat mixtures, solid ingredients were first weighed and then combined with the liquids. The mixture was blended using a Thermomix TM6 food processor (Vorwerk, Wuppertal, Germany) at medium-high speed for 3 min at room temperature. Each formulation was independently prepared in duplicate.

Subsequently, alternating layers of lean and fat phases were dosed into 26.5 × 13.5 × 6.5 cm molds, following a five-layer sequence with 60 g of lean phase and 30 g of fat phase per layer. The layering always began and ended with the lean formulation, resulting in a final ratio of 75% lean to 25% fat. The analogs were cooked in a Rational iCombi Pro oven (Rational AG, Landsberg am Lech, Germany) at 110 °C and 100% relative humidity. An integrated six-point immersion temperature probe was used to monitor the internal temperature to ensure each product reached 95 °C.

After baking, the salmon analogs were left to cool at room temperature for 1 h and were then refrigerated for 24 h. The next day, the samples were sliced lengthwise into 2 mm-thick slices using a EUROCORT semi-automatic slicer, model AF350INGR (Swedlinghaus, Ozzano dell’Emilia, Italy). For further evaluation, circular samples were obtained using a 20 mm diameter corer.

### 2.3. Technological Suitability: Sliceability and Layer Definition

The technological suitability of the gelled blocks was assessed based on two key aspects: sliceability and layer definition.

Sliceability was defined as the maintenance of structural integrity and firmness of the blocks while slicing with an automatic slicer. A scale from 0 to 3 was applied (0: impossible to slice, 1: incomplete or fragile slices, 2: acceptable slices with minor fragility, and 3: optimal slicing performance).

**Figure 2:**
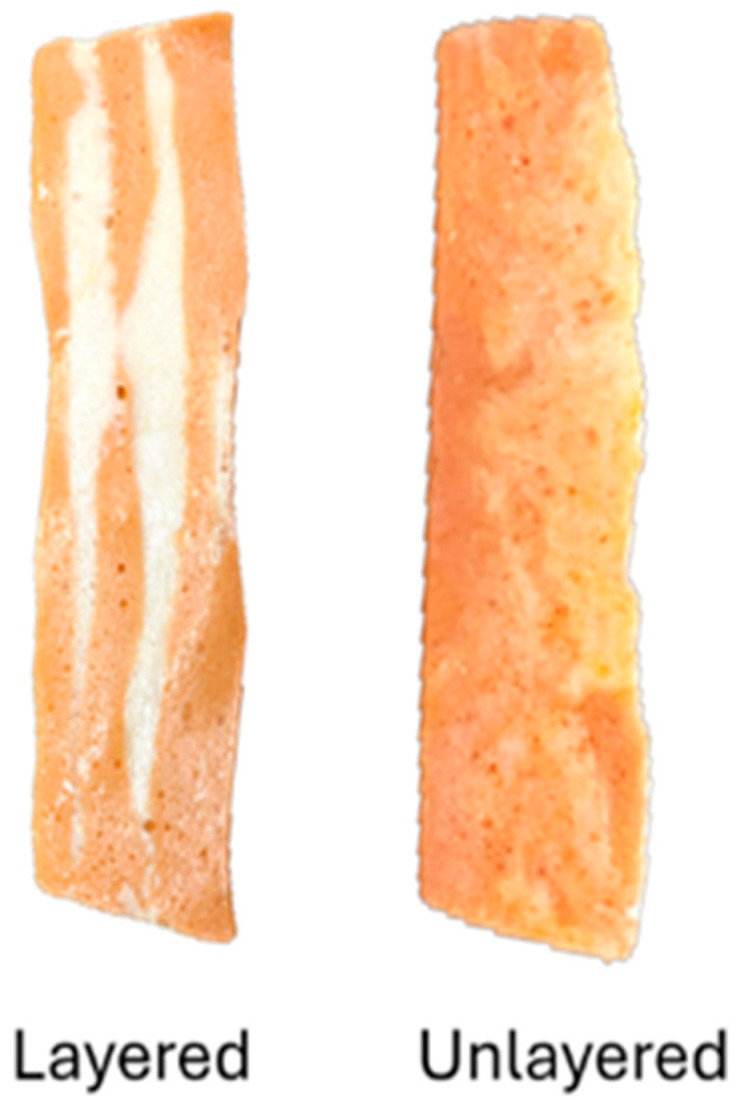
Degree of layer definition in salmon analog slices. **Left**: Sample with well-defined stratification. **Right**: Sample without visible layer separation.

Layer definition was defined as the ability of the block to maintain clear separation between lean and fat layers, avoiding mixing during gelation and slicing. The structural integrity and the visual clarity were both considered critical quality attributes for product evaluation (Figure 2).

### 2.4. pH

For pH determination, samples were prepared as cylindrical plugs to ensure consistent contact with the pH sensor (GLP 22 pH meter, Crison, Barcelona, Spain). The equipment was calibrated using buffer solutions at pH 7.00 and 4.00. Three measurements were taken at diverse points in each sample.

### 2.5. Water Binding Capacity (WBC)

Following the method of Wu et al. [21] with modifications, WBC was measured in two stages. In the first stage, 20 mm diameter discs were weighed using a TP 214 precision balance (Denver Instrument, Arvada, CO, USA). Each disc was then placed in an 8 mm ⌀ by 15 mm long, paper lined, Eppendorf tube containing a cotton filter. Samples were centrifuged at 3000× *g* for 10 min using a VWR Micro Star 17 centrifuge (VWR International, Radnor, PA, USA). After centrifugation, the discs were weighed again to calculate water loss.

In the second stage, the centrifuged discs were placed on pre-weighed, identified aluminum foil supports and dried in a Binder oven ED 23 (Binder GmbH, Tuttlingen, Germany) at 105 °C for 24 h to ensure complete dehydration. The dried discs were then cooled for 10 min in a desiccator containing silica gel and reweighed. WBC was calculated using Equation (1):

Water Binding Capacity (WBC): (E − C)/E × 100(1)

where:

A: initial sample weight (g)
B: weight after centrifugation (g)
C: water lost after centrifugation: A − B (g)
D: final dry weight (g)
E: water content of the sample: A − D (g).

### 2.6. Texture Profile Analysis (TPA)

Texture analysis was performed using cylindrical samples (20 mm ⌀ × 2 mm tall), with three replicates per formulation. Hardness and springiness were measured using a TA.XT2 Texture Analyser (Stable Micro Systems, Surrey, UK), equipped with a flat cylindrical probe (36 mm diameter). The test parameters were as follows: pre-test speed of 5 mm/s, test speed of 1 mm/s, post-test speed of 5 mm/s, a deformation of 50%, and a 5 s interval between compressions. The analyzer was calibrated prior to each session to ensure accuracy.

### 2.7. Rheological Characteristics

Rheological properties were assessed using an Anton Paar MCR 102e rheometer (Graz, Austria) with a plate–plate configuration and Peltier temperature control (20 ± 0.2 °C). The discs (20 mm ø by 2 mm thick) were slightly compressed between the plates, leaving a 1.5 mm gap. To determine the linear viscoelastic region (LVER), an amplitude sweep from 0.1% to 100% strain was first performed on a representative sample, following the method of Ran et al. [7]. Subsequently, frequency sweeps (0.1 to 100 Hz) were conducted on fresh, undamaged samples at 0.5% strain, which is within the previously determined LVER. The elastic modulus (G1), viscous modulus (G2), complex viscosity (η*) and phase angle (δ) were recorded; all parameters were analyzed specifically at 1 Hz.

### 2.8. Statistical Analysis

One-way analysis of variance (ANOVA) and Tukey’s post hoc test were used to assess statistical significance among treatments at a 95% confidence level (*p* < 0.05). All analyses were performed using Statgraphics Centurion XV (Statpoint Technologies, Inc., Warrenton, VA, USA).

Principal component analysis (PCA) was carried out to explore the relationships between textural and physicochemical attributes across the different formulations. Also using Statgraphics Centurion XV, Hierarchical cluster analysis (HCA) was performed to classify the formulations based on variable similarity.

## 3. Results and Discussion

Based on the following results, the influence of distinct proportions of iota carrageenan, kappa carrageenan, and konjac gum on the physical and technological properties of the vegan salmon analog formulations is discussed in detail.

### 3.1. pH, WBC and Appearance

The pH values of all samples analyzed, including formulations T01 to T16, ranged from 5.4 to 7.5; the commercial control was at 6.2 ± 0.1 (Table 2). Formulations containing higher concentrations of kappa and iota carrageenan (T01 and T02) tended to show elevated pH values, whereas those with a greater proportion of konjac gum (T05, T12, and T03) exhibited a lower pH. This aligns with previous studies that attribute the acidifying effect of konjac gum to deacetylation under heating conditions [7]. Slight variations within the 5.4–7.5 range may influence the surface charge and electrostatic interactions between pea proteins and the hydrocolloids, which could, in turn, modulate the gelation kinetics and the firmness of the final network.

**Figure 3:**
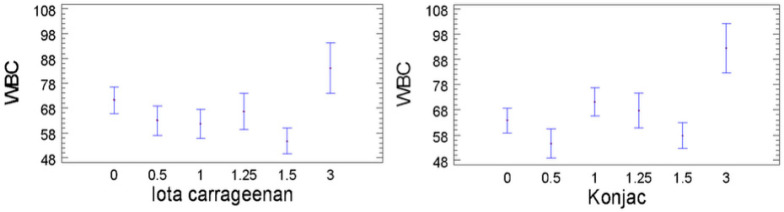
Effect of iota carrageenan (**left**) and konjac gum (**right**) concentrations on the water-binding capacity of salmon analog formulations. In all samples, the total hydrocolloid content was fixed at 3%, with the remaining proportion completed using the other polysaccharides (kappa carrageenan, iota carrageenan, and konjac gum, respectively) at concentrations of 0%, 0.5%, 1%, 1.25%, or 1.5%, as detailed in Table 1.

Regarding WBC, the control sample exhibited a high retention value of 87.4 ± 3.2%, placing it at the upper end of the observed spectrum (37.1% to 92.4%). Formulations T02 (high in iota carrageenan) and T03 (high in konjac gum) displayed the highest WBC values, which confirms the moisture-retaining ability of these individual hydrocolloids (Figure 3).

Konjac gum is known for its elevated water absorption and its capacity to convert free water into bound water within a three-dimensional matrix [7,22]. Majzoobi et al. [16], increased WBC in plant-based sausages when konjac gum was added. Other authors have noted that konjac forms extremely viscous aqueous solutions, even at low concentrations, and can retain up to 200 times its own weight in water [23].

Conversely, formulations with the lowest WBC, such as T06 and T13, contained lower levels of konjac gum. While individual use of iota or konjac gum may maximize WBC, their unbalanced combination may impair other technological attributes. Several formulations with more balanced blends exhibited reduced WBC but improved sliceability and layer definition. This suggests the requirement of an essential synergistic interaction among hydrocolloids to achieve optimal overall product properties.

Sliceability, defined as the ability to obtain thin, intact slices, was assessed on a 0 to 3 scale. Treatments T02 and T03 (high iota or konjac content with no kappa carrageenan) received the lowest scores (0), while T01 (exclusively kappa carrageenan) achieved the highest score (3). Formulations T04, T07, and T15 also presented low sliceability, consistent with their low kappa content. These results indicate that kappa carrageenan enhances the mechanical strength required for slicing.

Layer differentiation, referring to visual and structural separation between lean and fat phases, was only observed in six treatments: T04, T05, T07, T11, T12, and T16. All but T04 also had good sliceability. Among them, T16 was notable for combining near-control values for pH and WBC with well-defined layering and optimal slicing. In contrast, T14 and T15, despite presenting pH values similar to the control, exhibited significantly lower WBC and lacked visible layering, which indicates suboptimal structural performance. The absence of distinct layering in some samples is primarily attributed to differences in viscosity and density between the lean and fat mixtures, which affected the extent of phase separation during preparation.

Although viscosity measurements were not included in the present study, they represent a relevant parameter for understanding the behavior of the lean and fat phases during cooking. The observed partial mixing and layer definition suggest that differences in viscosity and density play a role in the final structure. Future studies should consider including viscosity profiling under thermal conditions to better characterize the rheological behavior of the full product matrix and optimize the formulation accordingly.

Furthermore, the lack of microstructural analysis represents another limitation of the current work. Microstructural visualization techniques, such as light microscopy or scanning electron microscopy (SEM), could offer valuable insights into the internal organization of the product, especially in relation to layer integrity and water distribution. These tools will be incorporated in future studies to further elucidate the mechanisms behind the texture and water-binding properties of plant-based salmon analogs.

### 3.2. Texture and Rheology

Texture Profile Analysis (TPA) revealed considerable variability across treatments in both hardness and springiness; with hardness values ranging from 4.6 N to 30.9 N and springiness from 2.1% to 90.6% (Table 3).

**Figure 4:**
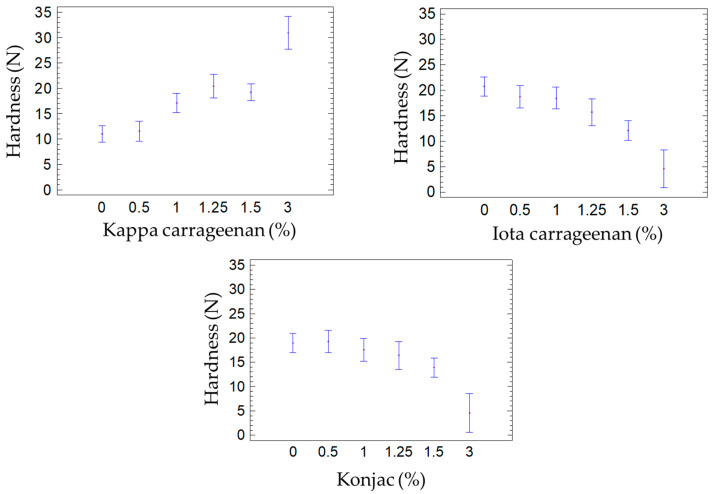
Mean and standard deviation of hardness values for salmon analog formulations as a function of individual gelling agents: kappa carrageenan (**top left**), iota carrageenan (**top right**), and konjac gum (**bottom**). Each plot groups the 16 formulations by the concentration of the corresponding hydrocolloid (0%, 0.5%, 1%, 1.25%, 1.5%, or 3%). In all samples, the total hydrocolloid content was fixed at 3%, with the remaining percentage made up of the other two hydrocolloids in varying proportions, as detailed in Table 1. *Y*-axis: Hardness (N). *X*-axis: Hydrocolloid concentration (%).

On the one hand, T01, containing a high proportion of kappa carrageenan, exhibited the highest hardness value (30.9 ± 3.3 N), exceeding that of the control (26.8 ± 6.8 N). This supports previous reports that kappa carrageenan forms rigid, dense gels [16,18]. Similarly, Verbeken et al. [24], observed increased hardness in meat products with added kappa carrageenan. On the other hand, T02 and T03, based on iota carrageenan and konjac gum, respectively, presented the lowest hardness values (4.6 ± 0.5 N), suggesting the formation of weaker and more elastic or hydrated networks (Figure 4).

Some studies suggest that konjac gum can significantly enhance hardness when deacetylated; however, the low values observed in this study imply that extensive deacetylation was not favored in the processing conditions applied [7]. In such systems, increasing the concentration of konjac glucomannan (KGM) and applying strong alkaline or thermal treatments lead to the removal of acetyl groups. This structural change causes the konjac chains to shift from a “semi-wrinkled” configuration to a “self-crimping” conformation, which promotes aggregation of the KGM molecules and the formation of more compact three-dimensional gel networks, thereby increasing the overall hardness of the product.

This discrepancy with the prior literature may be attributed to several influencing factors, including konjac concentration, initial pH, and the presence of salts or cations that affect network formation. In our case, the likely absence of deacetylation resulted in looser, less cohesive gel structures, leading to reduced mechanical strength. Moreover, the lack of information from the supplier regarding the degree of acetylation limits a deeper mechanistic understanding and highlights the need for further structural characterization in future work.

Regarding springiness, the T08 formulation (iota + konjac) presented the highest value (90.6 ± 2.9%), followed by T07 (87.5 ± 5.0%). This behavior can be attributed to the elastic characteristics of iota carrageenan and the moisture-retention effect of konjac gum [14,17]. In contrast, T03 (only konjac gum) displayed the lowest springiness (2.1 ± 0.4%), indicating that konjac alone may not support elastic recovery and could negatively affect sensory perception.

TPA provides valuable insights into sensory perception and is useful for quality control; however, its results are highly dependent on test conditions and do not offer precise information about the internal structure of the sample. In contrast, small-strain oscillatory tests can reveal the viscoelastic behavior of gels and provide critical data on their structure, making them indispensable tools for the assessment of mechanical and structural properties [7].

T01 stood out again with the highest elastic (29,311 ± 7259 Pa) and viscous (5839 ± 1363 Pa) moduli, both exceeding those of the control (14,064 ± 4799 Pa and 3675 ± 2290 Pa, respectively). These values indicate a stiff and cohesive gel matrix dominated by kappa carrageenan. In contrast, the lowest values (1046 ± 410 Pa for elastic modulus and 207 ± 75 Pa for viscous modulus) were recorded for T04 (iota + konjac, without kappa), suggesting a more fragile and deformable gel matrix. The literature indicates that konjac gum significantly increases viscosity only above specific concentration thresholds [23], which may not have been reached in this formulation.

Together, these findings highlight that gel strength and elasticity in plant-based analogs are not solely dependent on the individual hydrocolloids, but on their interactions and balance within the system. Synergistic combinations, particularly those involving all three hydrocolloids in optimal ratios, appeared more capable of replicating the mechanical behavior of real smoked salmon.

### 3.3. Principal Component Analysis (PCA)

**Figure 5:**
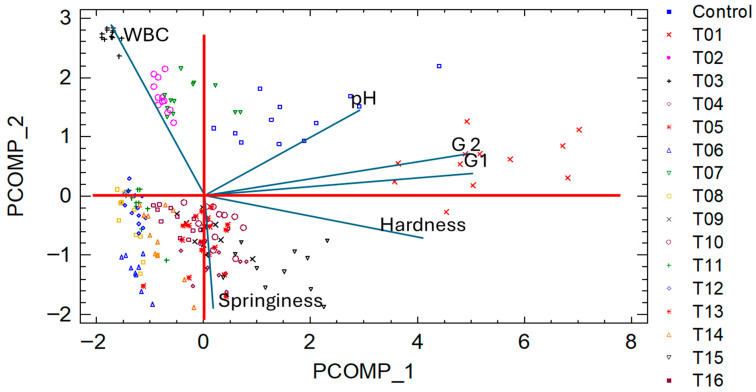
Principal Component Analysis (PCA) of physicochemical and textural attributes across 16 plant-based salmon analog formulations.

As shown in Figure 5, the PCA yielded two principal components based on the physicochemical and textural properties evaluated across the 16 salmon analog formulations; together they accounted for 72.52% of the total variance. Component 1 (PCOMP_1) was primarily associated with mechanical attributes such as hardness, elastic modulus (G1), and viscous modulus (G2), which showed the highest positive contributions, explaining the main differentiation between formulations along this axis. Conversely, water-binding capacity (WBC) exhibited a significant negative loading. This indicates an inverse relationship between structural rigidity and water retention, consistent with the trends previously described for individual texture and rheological results. PCOMP_2 was mainly associated with springiness, opposing WBC, which reinforces the contrasting behavior of springiness versus moisture content within the gels.

The spatial distribution of the formulations in the PCA biplot clearly reflects the relationships among the mechanical and physicochemical attributes. The commercial control sample, situated in the positive quadrant of PCOMP_1, corresponds to high values of hardness and modulus, reflecting a firm and structurally resistant matrix. Formulations T01 and T13 were positioned near the control along this axis, indicating similar mechanical behavior, particularly in terms of structural strength.

T02 appeared closest to the control along PCOMP_2, implying a comparable balance of springiness and WBC. However, the markedly lower position along PCOMP_1 indicates reduced mechanical firmness, relative to the control. This suggests that although T02 mimics the control in terms of springiness and water retention, it lacks structural robustness.

In contrast, T16 occupied a position close to the control on PCOMP_2 and a moderately negative location on PCOMP_1. This indicates that its mechanical properties were not as firm as the control; its balance between springiness and WBC more closely resembled that of the reference product than other formulations positioned further away from the origin. These associations confirm that formulations containing balanced proportions of the three hydrocolloids achieved a closer mechanical and structural resemblance to the control product.

Although T13 closely matched the control in terms of hardness and modulus (PCOMP_1), it scored negatively on PCOMP_2, indicating low springiness and high WBC. This combination may result in a more hydrated and less elastic texture, deviating from the intended sensory profile.

Finally, formulations such as T14 and T15, located in the lower left quadrant of the PCA biplot (negative on both components), exhibited low hardness, low springiness, and high retention of water, indicating a profile substantially different from that of the commercial control.

**Figure 6:**
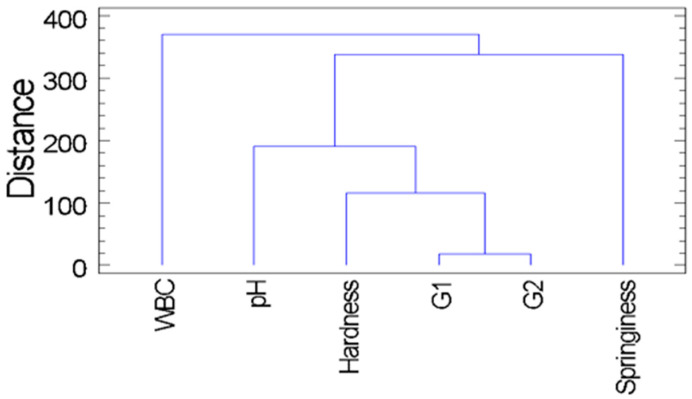
Hierarchical Cluster Analysis (HCA) dendrogram based on physicochemical and textural properties of plant-based salmon analog formulations.

As shown in Figure 6, the hierarchical cluster analysis (HCA) enabled the classification of physicochemical and textural variables into two main clusters.

The first cluster consisted solely of water-binding capacity (WBC), while the second cluster grouped the remaining variables: pH, hardness, elastic modulus (G1), viscous modulus (G2) and springiness. Within this second cluster, two distinct subgroups were identified. The first subgroup comprised pH, hardness, G1 and G2, with G1 and G2 showing the greatest similarity (shortest linkage distance). This short linkage distance suggests highly correlated behavior. Hardness was linked to this pair, followed by pH at a greater distance, indicating slightly more independent behavior.

The second subgroup was composed exclusively of springiness, which branched separately from the first group of mechanical parameters, reflecting a distinct behavioral pattern. This structure suggests that, although springiness is a mechanical attribute, its behavior differs from hardness and modulus values, which may respond differently to formulation variables.

The HCA results were consistent with those of the PCA, which also demonstrated a clear distinction between springiness and the other mechanical variables. In both analyses, WBC was negatively associated with structural firmness thus grouped independently, reinforcing the interpretation that water retention tends to counteract gel strength.

Although pH presented a closer relationship to hardness and the elastic and viscous moduli, it retained a degree of independence while occupying an intermediate position in the cluster. This suggests that although it contributes to the mechanical profile, its effect is less directly aligned with textural strength than other variables.

Overall, these results support the presence of two primary groups of variables: one associated with structural and firmness-related properties (hardness, elastic modulus, viscous modulus and pH), and another consisting of springiness and water retention (springiness and WBC), which exhibited distinct behaviors in the clustering analysis.

## 4. Conclusions

This work demonstrates that a strategic combination of kappa carrageenan, iota carrageenan, and konjac gum enables the development of plant-based salmon analogs with tailored textural and structural properties. The functional differences observed can be attributed to the distinct gelling behaviors and molecular mechanisms of each hydrocolloid. Kappa carrageenan was essential for achieving firmness and structural integrity, improving sliceability and providing mechanical resistance comparable to conventional smoked salmon due to its ability to form strong, cohesive networks through helix aggregation. Iota carrageenan enhanced elasticity and visual layering, contributing to a softer and more flexible texture as a result of its highly hydrated and elastic gel structure. Konjac gum supported water retention and internal juiciness through its viscous matrix, although excessive levels were found to compromise firmness and sliceability by weakening the gel network.

These findings confirm that the mechanical and moisture-related performance of the formulations depends not only on the individual properties of each hydrocolloid, but also on their relative ratios and interactions. Overall, this study provides a robust framework for the rational design of alternative seafood products with optimized texture, juiciness and structural definition.

Multivariate analyses reinforced these observations by identifying two main clusters of correlated variables: one associated with mechanical strength (hardness, elastic modulus, viscous modulus, and pH), and another linked to springiness and water-binding capacity. This division underscores the dual technological functionality required to mimic the texture of animal-based smoked salmon.

Among the 16 tested formulations, T01, T02, and T16 emerged as the most promising prototypes, each representing a distinct but relevant textural profile. T01, formulated with 3% kappa carrageenan, exhibited mechanical firmness and sliceability closely resembling the commercial control, making it suitable for applications requiring structural stability. T02, containing 3% iota carrageenan, demonstrated superior springiness and water retention, ideal for softer, juicier products. T16, composed of equal proportions (1%) of all three hydrocolloids, achieved a well-balanced profile combining firmness, elasticity, and visual definition, highlighting the synergistic potential of these ingredients.

These findings confirm that the mechanical and moisture-related performance of the formulations depends not only on the individual properties of each hydrocolloid, but also on their relative ratios and interactions. Overall, this study provides a robust framework for the rational design of alternative seafood products with optimized texture, juiciness, and structural definition.

## Figures and Tables

**Table 1:** Percentage of hydrocolloids added to each formulation for thickening purposes.

	T01	T02	T03	T04	T05	T06	T07	T08	T09	T10	T11	T12	T13	T14	T15	T16
KCa	3.00	0.00	0.00	0.00	1.50	1.50	0.50	0.50	0.50	1.50	1.25	1.00	1.50	1.25	1.00	1.00
ICa	0.00	3.00	0.00	1.50	0.00	1.50	1.00	1.25	1.50	0.50	0.50	0.50	1.00	1.25	1.50	1.00
KGu	0.00	0.00	3.00	1.50	1.50	0.00	1.50	1.25	1.00	1.00	1.25	1.50	0.50	0.50	0.50	1.00

KCa: kappa carrageenan; ICa: iota carrageenan; KGu: konjac gum.

**Table 2:** pH, water binding capacity (WBC), sliceability, and layer differentiation of the 16 salmon analog formulations, varying the proportions of kappa carrageenan, iota carrageenan, and konjac gum are included. Values are presented as means ± standard deviation.

Treatment	WBC	pH	Sliceability	Layers
	(%)		0–3	Yes-No
Control	87.4 ± 3.2 ^hi^	6.2 ± 0.1 ^f^	3	Yes
T01	46.6 ± 8.4 ^abc^	7.5 ± 0.1 ^h^	3	No
T02	84.0 ± 6.9 ^ghi^	7.3 ± 0.0 ^g^	0	No
T03	92.4 ± 3.7 ^i^	5.8 ± 0.1 ^c^	0	No
T04	41.0 ± 14.2 ^ab^	6.0 ± 0.1 ^de^	1	Yes
T05	53.4 ± 11.0 ^bcd^	5.6 ± 0.1 ^a^	3	Yes
T06	37.2 ± 10.2 ^a^	6.0 ± 0.1 ^de^	2	No
T07	74.2 ± 5.9 ^fgh^	6.0 ± 0.0 ^de^	2	Yes
T08	71.6 ± 12.3 ^efg^	5.9 ± 0.1 ^cd^	3	No
T09	74.8 ± 10.7 ^fgh^	6.0 ± 0.1 ^de^	3	No
T10	64.9 ± 4.1 ^def^	6.1 ± 0.1 ^ef^	3	No
T11	63.8 ± 9.2 ^def^	6.1 ± 0.1 ^ef^	3	Yes
T12	60.2 ± 17.3 ^cde^	5.6 ± 0.1 ^b^	3	Yes
T13	37.1 ± 11.5 ^a^	6.0 ± 0.0 ^cde^	3	No
T14	61.7 ± 7.8 ^def^	6.2 ± 0.0 ^f^	3	No
T15	65.0 ± 7.9 ^def^	6.2 ± 0.1 ^f^	1	No
T16	73.9 ± 6.4 ^fgh^	6.1 ± 0.1 ^ef^	3	Yes

A different letter in the same column indicates a significant difference as determined by the Tukey test (*p* < 0.05).

**Table 3:** Textural and viscoelastic parameters of the vegan salmon analog formulations: hardness, springiness, elastic modulus, and viscous modulus.

	Hardness	Springiness	Elastic Modulus	Viscous Modulus
Treatment	N	%	Pa	Pa
Control	26.8 ± 6.8 ^g^	55.9 ± 2.8 ^c^	14,064 ± 4799 ^j^	3675 ± 2290 ^e^
T01	30.9 ± 3.3 ^h^	71.2 ± 2.8 ^e^	29,311 ± 7259 ^k^	5839 ± 1363 ^f^
T02	4.6 ± 0.5 ^a^	63.3 ± 5.6 ^d^	3920 ± 535 ^bcd^	757 ± 109 ^abc^
T03	4.6 ± 0.6 ^a^	2.1 ± 0.4 ^a^	4209 ± 700 ^cde^	1100 ± 147 ^abc^
T04	7.9 ± 1.8 ^ab^	79.2 ± 8.2 ^f^	1046 ± 410 ^a^	207 ± 75 ^a^
T05	20.7 ± 2.5 f	80.1 ± 5.6 ^f^	7620 ± 2364 ^hi^	1305 ± 373 ^bc^
T06	13.5 ± 1.2 ^cde^	81.2 ± 1.9 f	9038 ± 1844 ^i^	1671 ± 360 ^cd^
T07	11.8 ± 1.4 ^cd^	87.5 ± 5.0 ^gh^	1969 ± 538 ^ab^	402 ± 116 ^ab^
T08	11.3 ± 1.2 ^bc^	90.6 ± 2.9 ^h^	1943 ± 588 ^ab^	397 ± 111 ^ab^
T09	11.5 ± 1.1 ^bc^	88.4 ± 3.4 ^gh^	2926 ± 709 ^abc^	563 ± 154 ^ab^
T10	20.0 ± 1.9 ^f^	85.4 ± 2.3 ^fgh^	6647 ± 1524 ^gh^	957 ± 220 ^abc^
T11	21.1 ± 1.5 ^f^	87.9 ± 3.5 ^gh^	6562 ± 2154 ^fgh^	1027 ± 303 ^abc^
T12	15.2 ± 1.4 ^de^	82.6 ± 3.1 ^fg^	5184 ± 1446 ^defg^	840 ± 224 ^abc^
T13	22.8 ± 1.8 ^f^	54.4 ± 6.8 ^fg^	12,335 ± 3463 ^j^	2552 ± 927 ^d^
T14	19.7 ± 1.4 f	85.0 ± 3.1 ^fgh^	7903 ± 184 ^hi^	1248 ± 284 ^bc^
T15	15.4 ± 1.7 ^e^	88.6 ± 2.0 ^gh^	4464 ± 1661 ^bcdef^	793 ± 273 ^abc^
T16	20.8 ± 2.8 ^f^	13.5 ± 1.6 ^b^	6264 ± 2973 ^efgh^	890 ± 414 ^abc^

Mean values and standard deviation (n = 12). A different letter in the same column indicates a significant difference determined by the Tukey test (*p* < 0.05).

## Data Availability

The data presented in this study are available on request from the corresponding author.

## Abbreviations

ANOVA	One-way analysis of variance
G1	elastic modulus
G2	viscous modulus
GRAS	Generally Recognized as Safe
HCA	Hierarchical cluster analysis
KGM	Konjac glucomannan
LVER	linear viscoelastic region
PCA	Principal component analysis
PCOMP_1	Component 1
PCOMP_2	Component 2
T	Treatment
TPA	Texture Profile Analysis
WBC	Water Binding Capacity
